# The seasonal influence of climate and environment on yellow fever transmission across Africa

**DOI:** 10.1371/journal.pntd.0006284

**Published:** 2018-03-15

**Authors:** Arran Hamlet, Kévin Jean, William Perea, Sergio Yactayo, Joseph Biey, Maria Van Kerkhove, Neil Ferguson, Tini Garske

**Affiliations:** 1 MRC Centre for Outbreak Analysis and Modelling, Department of Infectious Disease Epidemiology, Imperial College London, London, United Kingdom; 2 Laboratoire MESuRS, Conservatoire National des Arts et Métiers, Paris, France; 3 WHO, Infectious Hazard Management, Geneva, Switzerland; 4 WHO-AFRO, IST/WA, Ouagadougou, Burkina, Faso; 5 Centre for Global Health, Institut Pasteur, Paris, France; Saudi Ministry of Health, SAUDI ARABIA

## Abstract

**Background:**

Yellow fever virus (YFV) is a vector-borne flavivirus endemic to Africa and Latin America. Ninety per cent of the global burden occurs in Africa where it is primarily transmitted by *Aedes spp*, with *Aedes aegypti* the main vector for urban yellow fever (YF). Mosquito life cycle and viral replication in the mosquito are heavily dependent on climate, particularly temperature and rainfall. We aimed to assess whether seasonal variations in climatic factors are associated with the seasonality of YF reports.

**Methodology/Principal findings:**

We constructed a temperature suitability index for YFV transmission, capturing the temperature dependence of mosquito behaviour and viral replication within the mosquito. We then fitted a series of multilevel logistic regression models to a dataset of YF reports across Africa, considering location and seasonality of occurrence for seasonal models, against the temperature suitability index, rainfall and the Enhanced Vegetation Index (EVI) as covariates alongside further demographic indicators. Model fit was assessed by the Area Under the Curve (AUC), and models were ranked by Akaike’s Information Criterion which was used to weight model outputs to create combined model predictions. The seasonal model accurately captured both the geographic and temporal heterogeneities in YF transmission (AUC = 0.81), and did not perform significantly worse than the annual model which only captured the geographic distribution. The interaction between temperature suitability and rainfall accounted for much of the occurrence of YF, which offers a statistical explanation for the spatio-temporal variability in transmission.

**Conclusions/Significance:**

The description of seasonality offers an explanation for heterogeneities in the West-East YF burden across Africa. Annual climatic variables may indicate a transmission suitability not always reflected in seasonal interactions. This finding, in conjunction with forecasted data, could highlight areas of increased transmission and provide insights into the occurrence of large outbreaks, such as those seen in Angola, the Democratic Republic of the Congo and Brazil.

## Author summary

In this article, we describe the development of a model to quantify the seasonal dynamics of yellow fever virus (YFV) transmission across Africa. YFV is a flavivirus transmitted, within Africa, primarily by *Aedes spp* where it causes an estimated 78,000 deaths a year despite the presence of a safe and effective vaccine. The importance of sufficient vaccination, made difficult by a global shortage, has been highlighted by recent large scale, devastating, outbreaks in Angola, the Democratic Republic of the Congo and Brazil. Here we describe a novel way of parameterising the effect of temperature on YFV transmission and implement statistical models to predict both the geographic and temporal heterogeneities in transmissions, while demonstrating their robustness in comparison to models simply predicting geographic distribution. We believe this quantification of seasonality could lead to more precise applications of vaccination campaigns and vector-control programmes. In turn this would help maximise their impact, especially vital with limited resources, and could contribute to lessening the risk of large scale outbreaks. Not only this, but the methods described here could be applied to other *Aedes*-borne diseases and as such provide a useful tool in understanding, and combatting, several other important diseases such as dengue and zika.

## Introduction

Yellow fever (YF) is a viral disease caused by the mosquito transmitted yellow fever virus (YFV) of the genus Flavivirus [1]. Though endemic to both Africa and South America it is thought that 90% of cases occur in Africa [2], where the virus causes between 51,000 and 380,000 severe cases per year resulting in around 19,000 to 180,000 deaths [3]. Roughly half of those infected experience an asymptomatic infection and a further third a mild illness, however, about 12% of YF infections progress to severe disease, defined as fever with jaundice and/or haemorrhage, with a mortality rate of 30–60% [4]. In Africa the disease is found in three transmission cycles: (i) a sylvatic cycle where transmission is maintained between non-human primates via sylvatic *Aedes* mosquitoes, such as *Ae*. *africanus*, that gives way to (ii) an intermediate cycle where peri-domestic *Aedes* species act as a bridging point between humans and non-human primates. This can lead to the establishment of (iii) an urban cycle propagated by *Aedes aegypti* [2, 5]. In this urban cycle the disease is spread between humans and can establish itself in areas without a sylvatic host. Historically, this has led to devastating epidemics which reached as far as The United States of America, England and Italy during the 16^th^-19^th^ century [6].

The primary vector species for YFV transmission in humans is the mosquito *Ae*. *aegypti* [7], which carries several other human viruses such as dengue and chikungunya [8, 9]. *Ae*. *aegypti*, like all insects, is a poikilothermic ectotherm which means that its internal temperature is linked to the ambient temperature. As temperature increases, so does metabolism. This has a wide variety of implications for the transmission of YFV. Higher temperatures, below temperature induced mortality, increase the speed of pupation [10], raise the competence of the immune system [11], the frequency of blood meals [12] and reduce the extrinsic incubation period (EIP) of YFV, defined as the time from a mosquito acquiring infection to becoming infectious [13]. However, temperature is neither the sole determinant of *Ae*. *aegypti* natural history, nor a standalone factor. The interplay between precipitation and temperature may be more important than either variable alone: both optimal temperatures with insufficient rainfall and insufficient temperatures with optimal rainfall are unfavourable for *Ae*. *aegypti*, and viral replication within the mosquito, therefore limiting the potential for transmission.

Previously, several models have used climatic factors in order to map the distribution and transmission of YF. While Rogers et al., (2006) [14] modelled the global distribution of YF using only satellite derived environmental data, Garske et al., (2014) [3] used human population sizes, surveillance quality and geographical coordinates, along with environmental data to predict the distribution of YF in Africa. This was then used in conjunction with serological surveys in order to generate the force of infection across the endemic zone and, taking into account vaccination coverage, the burden of disease. In addition to YF, the distribution and transmission of several other vector-borne diseases such as malaria [15–19] and dengue [20–23] have been modelled using climatic factors.

Not only have static variables such as annual temperature and rainfall been shown to have substantial predictive ability, but the importance of seasonality has also been highlighted [16, 19, 24], suggesting that taking into account seasonality in disease control and prevention interventions could translate to substantial public health gains [16]. Characterising the relationship between YF occurrence and environmental factors, in combination with future predictions of these factors, could allow for forecasting of periods of “heightened risk”. These predictions would then allow proactive intensification of surveillance to detect initial cases and respond with appropriate measures such as vaccination or vector-control. Despite previous success in utilising climate and seasonality to evaluate disease dissemination and intensity, this approach has not previously been applied to YF. The importance of further quantifying the epidemiology behind YFV transmission has been highlighted in recent years by large scale outbreaks in Angola, the Democratic Republic of the Congo and Brazil [25, 26].

The aim of this study was to investigate the effect of climate and the role of seasonality in YFV transmission using a limited number of temporally varying covariates, in contrast to the initial stage of the Garske et al., (2014) [3] which used a wide array of static environmental and non-environmental covariates. To this end we developed a temperature suitability index for the transmission of YFV by *Ae*. *aegypti* which captures our understanding of the temperature dependence of the mosquito life cycle and viral replication mechanistically, following similar approaches used for malaria and dengue [8, 17]. The predictive power of this index was assessed alongside other variables by fitting a series of multilevel logistic regression models to YFV occurrence across Africa to capture both geographical and seasonal variation.

## Methods

### Datasets

Disease occurrence in a province was defined as at least a single laboratory confirmed YF case. An occurrence dataset was compiled from various sources, including YF reports between 1971 and 2015 from the Weekly Epidemiological Record [27] and the Disease Outbreak News [28], as well as individual reports of laboratory confirmed cases between 2007 and 2013 from the Yellow Fever Surveillance Database, see Garske et al., (2014) [3].

Each of the reports were geo-located to the first sub-national administrative level, here termed province, and provinces across Africa were classified into the presence or absence of any confirmed YF reports from any data source over the study period. Furthermore, we generated a seasonal dataset, where we similarly classified each province and calendar month into YF presence or absence, based on the start month of the outbreak or individual reported case.

Surveillance quality was informed by the volume of reporting suspected YF cases into the Yellow Fever Surveillance Database (YFSD). This collects data on suspected cases of YF using a very broad case definition based on the syndrome fever and jaundice, of which only 1–2% are laboratory confirmed while the remaining suspected cases, which form the vast majority, are due to other causes. Assuming an approximately constant incidence of the syndrome “fever and jaundice” across the region covered, we used the per-capita rate of reporting suspected cases as a proxy for the surveillance quality in participating countries. With non-participating countries assigned the mean value.

Country and year specific population sizes were obtained from the UN World population prospects (UN WPP 2014) [29] and then averaged over the study period (1971–2015) to obtain average population sizes. Population sizes were disaggregated to the first sub-national administrative unit, termed province, by aggregating the LandScan 2011 [30] population estimates from a 1km grid [3] to the province level to calculate the proportion of the national population within each province. This proportion was assumed to be constant through time to calculate the year-specific population sizes by province from the UN WPP national population sizes.

Datasets for seasonal air temperature [17], the enhanced vegetation index (EVI), an optimised satellite derived measure of vegetation, [31] and rainfall [32], from 2003 to 2006, the period for which all this data was available, were used as covariates. These were chosen a priori based on the ability to plausibly explain their influence on transmission mechanistically and adoption in models from literature. This data was available in grids at a resolution of between 1 and 10km, which we aggregated to the province level, by calculating the population-weighted mean value, based on the spatial population distribution from the LandScan dataset [30], in order to generate environmental values that are representative of human habitation. While the timescale of this data is temporally mismatched with the case data, previous work by Garske et al., (2014) has found the approach taken here is a valid simplification of fitting to datasets that vary annually, not just seasonally. Given the uncertainty and sparsity of underlying case data the model complexity is appropriate considering the richness of data. All environmental datasets were stratified geographically to the province level and we used overall means through time as well as monthly and weekly values averaged across years to describe the typical seasonal patterns. The monthly and weekly datasets were obtained through Fourier transforms [17] to produce the smoothed and averaged outputs desired.

### Temperature suitability index and its components

The Ross-Macdonald model for mosquito borne disease transmission [33] defines the basic reproductive number, *R*_*0*_, as

$$R_{0}=\frac{ma^{2}bce^{-\mu EIP}}{\gamma \mu }.$$ [1]

Here, the vector to host ratio *m*, the probability of transmission between host and vector and vector and host in a single infectious bite, *b* and *c*, respectively and the recovery rate of the human host *γ*, are assumed to be temperature independent, while the mosquito mortality rate *μ*, the extrinsic incubation period *(EIP)* and the biting rate *a*, are assumed to be temperature dependent. The temperature suitability index *z*, is then defined as the temperature dependent factors of the basic reproduction number [8, 17, 18],

$$z(T)=\frac{a(T{)}^{2}e^{-\mu (T)EIP(T)}}{\mu (T)}.$$ [2]

This provides a single value parameterising the suitability for transmission at a given temperature *T*. The vector to host ratio, *m*, is likely to vary between locations and seasonally due to, among other factors, the abundance of breeding sites made available by rainfall. While the transmission probabilities may depend on the vector competence of local mosquito species, the human recovery rate is likely less variable geographically. Humans are only viraemic during the initial period of infection and not during the severe stage of disease [34].

### Data on the temperature dependence

**Fig 1:**
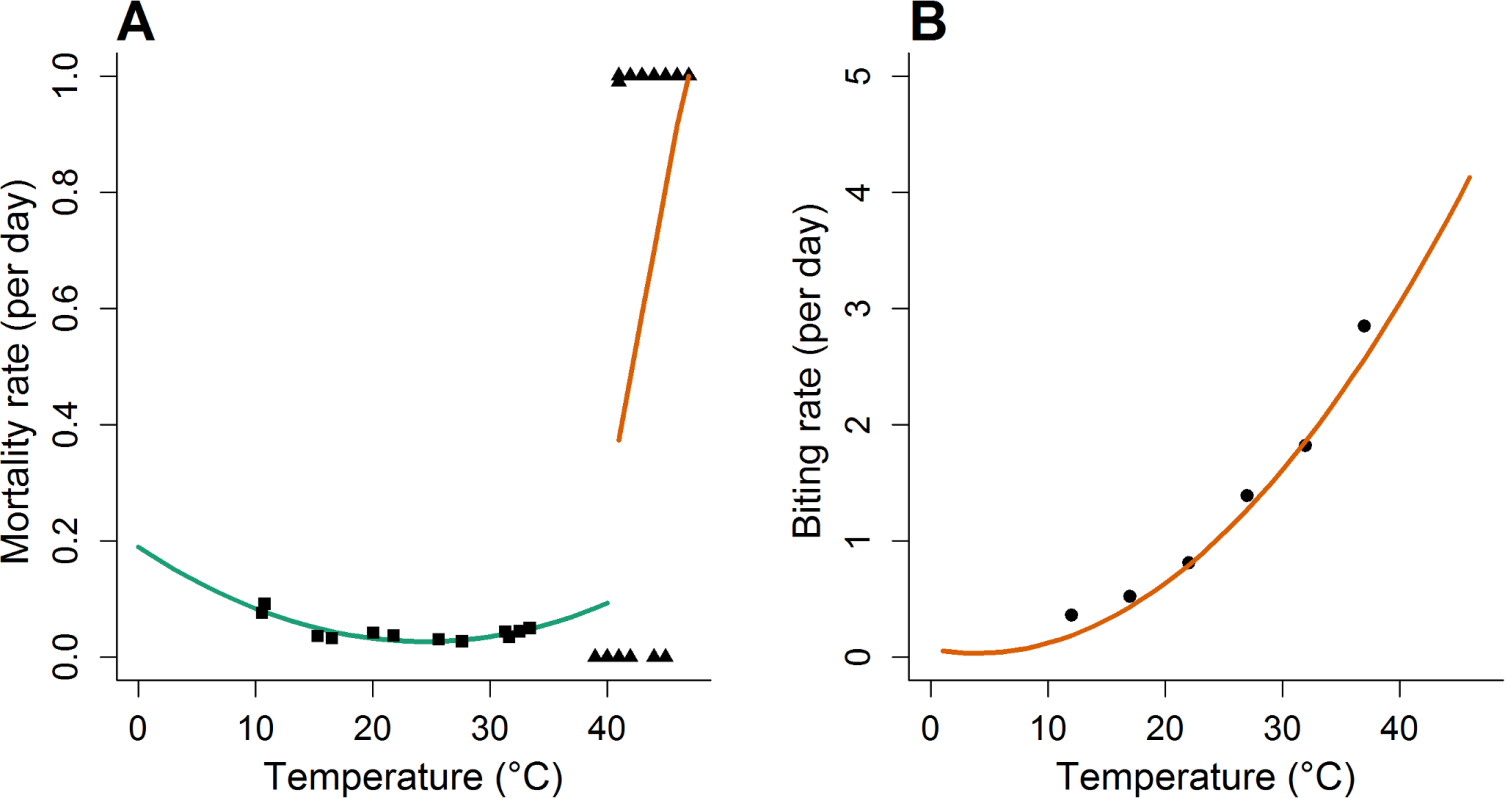
Daily temperature dependent morality and biting rate over a range of temperatures. A) Daily temperature dependent mortality, triangles indicate data from Christophers (1960)[36] and squares data from Yang et al., (2009)[35]. The lines depict the linear relationship between temperature and mortality below 41°C (green) and above 41°C (red). B) Daily biting rate against temperature from Martens (1998)[44]. Black dots show the data and the red line the polynomial fit.

The temperature dependent death rate *μ(T)*, was parameterised by fitting a piecewise linear relationship to mosquito mortality data for *Ae*. *aegypti* at different temperatures, with a cut point at 41°C [35, 36]. We fitted a linear regression to the data on the temperature dependence of mortality and biting rates (Fig 1). For mortality rates we fitted these separately for low (below 41°C) and high (above 41°C) temperature regimes, following exploratory analysis.

Assuming only one bite per gonotrophic cycle (i.e. the biting rate equals the oviposition rate) and that biting occurs solely on human hosts we fitted a linear relationship between temperature and biting rate to data on the temperature dependence of *Aedes* feeding using [37] a polynomial relationship.

The *EIP* shortens with increasing temperature, and the rate of turning infectious can be described by a certain number of degree-days required for viral replication [15, 38, 39]. For this we used the temperature dependent relationship described in Johansson et al., (2010)[13].

Suitable temperatures alone are not sufficient for mosquito survival and disease transmission; they need to coincide with rainfall in order to produce an optimal climate for transmission, as such the interaction between temperature suitability and rainfall was also considered as a potential covariate. In the seasonal version this was defined as the product of the monthly temperature suitability index and the rainfall during the previous month. This delay was implemented following exploratory analyses of the data, and may reflect the delayed impact rainfall has on the availability of mosquito breeding sites. To account for the seasonal synchronicity between temperature and rainfall we averaged the seasonal temperature suitability and rainfall interaction across the year for use in the annual model.

### Regression models

Seasonality was investigated using a series of multivariable models [40] fitted to the YF report dataset using a complementary log-log link function, with a variety of sets of candidate environmental covariates. This is conceptually similar to running a logistic regression with each data point referring to a province and calendar month. However, unlike in a logistic regression, multilevel models allow for parameters to vary by group, here the province, in order to avoid biases introduced by treating monthly covariates within a province as independent [41].

These models were fit to the whole continent of Africa. All models included the log_10_ of population size and the surveillance quality proxy for countries included within the YFSD [3], and the mean value of participating countries given to countries not included. Furthermore we included each possible subset out of the four considered environmental covariates, the temperature suitability index, rainfall, the interaction of temperature suitability and rainfall, and the EVI, resulting in 15 different models that each included at least one of the climatic covariates. Using these covariates we fitted multilevel logistic regression models to the presence/absence of YF reports by province in annual as well as seasonal models. Annual models were fitted to the overall presence/absence of YF reports using annual mean covariates, while seasonal models were fitted to the presence/absence of YF reports in each calendar month, using monthly varying covariates within each province. The seasonal models were fitted in a multilevel framework, where time points were nested in locations. In order to facilitate the comparison of model predictions between the seasonal and annual models we converted the predictions of the regression model quantifying the monthly probability of YF reports to annual values using

$$p_{year}=1-(\left(1-p_{1})(1-p_{2})\ldots (1-p_{12}\right)),$$ [3]

where *p*_*1*_ refers to January, *p*_*2*_ to February etc. We refer to these models as compound seasonal models.

Models were fitted using every possible combination of environmental covariates, yielding 15 different variations for the annual and seasonal models each. Models were scored based on Akaike Information Criterion (AIC) and the predictions from any model with an AIC less than 5 higher than the best performing model, as defined as the model with the lowest AIC value, were combined using Akaike weights [42]. This is achieved by computing the differences in AIC,

$$\Delta_{i}=AIC_{i}-\min\left(AIC\right),$$ [4]

which was then used to obtain an estimate of the relative likelihood of model *i*, in proportion to the other models *k* = 1 … *K* included in the combined estimate through,

$$w_{i}=\frac{exp\;\{-\frac{1}{2}\Delta_{i}\}}{\sum_{k=1}^{K}exp\;\{-\frac{1}{2}\Delta_{k}\}}.$$ [5]

This provided model specific weights, *w*_*i*_, which we applied to the predicted values of the specific model, *p*_*i*_, to generate a single set of Akaike weighted predictions, *p*_*A*_,

$$p_{A}=\sum_{k=1}^{K}{w_{k}p}_{k}.$$ [6]

For each set of covariates we assessed model fit using ROC (receiver operating characteristic) curves and the AUC (area under the curve) [42]. The ROC is a graphical plot which illustrates diagnostic ability of a test, with the AUC providing a numerical value of this ability. An AUC value of 0.5 indicates the diagnostic ability is no greater than random and a value of 1 as completely predictive.

The validity of predictions was ascertained through leave-one-out cross validation [43], where we divided the dataset by randomly assigning countries to one of five non-overlapping subsets, then fitted the models to the dataset omitting each of the subsets in turn to generate out-of-sample predictions for the omitted subset. This was repeated 10 times, resulting in 10 different allocations of provinces into subsets. For each province, the average was taken across the 10 realisations. Out-of-sample predictions for each subset were combined to generate a full set of out-of-sample predictions and its AUC compared with the full model.

All calculations and analyses were conducted in R version 3.2.5.

## Results

A linear model fit to temperature dependent mortality data provided by Yang et al., (2009) [35] predicts a mortality rate of 0.190 at 0°C which falls to a low of 0.027 at 25°C, before rising to 0.085 at 40°C. A model fit to data provided by Christophers (1960) [36] at higher temperatures shows a rapid increase from 0.380 at 41°C to 1.000 at 47°C (Fig 1A). A linear model fit to temperature dependent biting rate, from Martens (1998) [37], rises from 0 at 0°C to 4.5 at 47°C.

**Fig 2:**
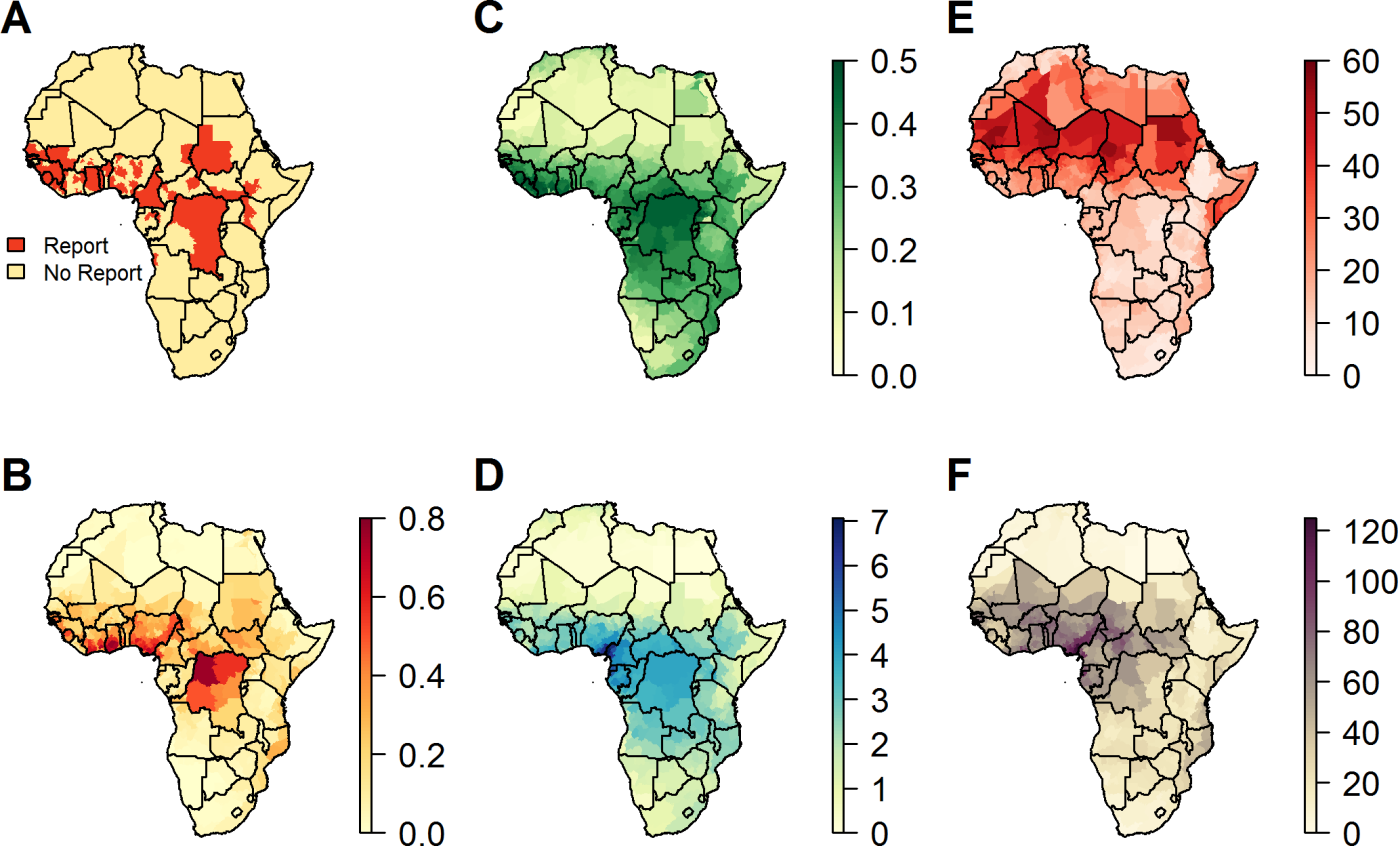
Annual covariates and report predictions. A) Presence/absence of YF reports (1971–2015) that fit inclusion criteria, B) model predictions from the annual model showing the probability of YF report from 1971–2015, C) mean annual EVI, D) mean annual rainfall, E) mean annual temperature suitability index and F) mean annual interaction of temperature suitability and rainfall.

We identified 167 unique province-months with YF reports for which the month of the report was known and could be identified at the province level, occurring in 105 unique provinces (Fig 2A). Model predictions of the probability of a YF report across Africa from the annual model reproduced the geographical distribution of YF reports well, with an AUC of 0.83 (95% CI 0.80; 0.87) (Fig 2B, see also fig in S3 Text for ROC curves of best performing models). The YF report probabilities range from 0 for much of Southern and Northern Africa, to above 0.70 in parts of the Democratic Republic of the Congo (DRC), Sierra Leone, Ghana and Côte d'Ivoire. East Africa has notably low report probabilities except in Sudan and South Sudan.

The spatial distribution of the mean annual covariates is shown in Fig 2C–2F. The areas with the highest mean annual temperature suitability index were found to be the Sahara and parts of the Horn of Africa (Fig 2E). Notably high index scores were found across West and parts of Central Africa, within the YF endemic zone [3]. The temperature suitability is lower throughout Northern and Southern Africa, as well as regions of higher elevation in East and Central Africa. Temperatures encountered in the Sahara appear highly suitable for transmission but the lack of rainfall is a limiting factor for transmission (Fig 2D). The largest values of the mean annual interaction of temperature suitability and rainfall (Fig 2F) are clustered around West and Central Africa, roughly approximating the regions where the burden of YF is the highest. North and South Africa have negligible levels throughout, and East Africa shows marginally raised levels in limited locations. These patterns are similar to the spatial distribution of the EVI (Fig 2C), although the interaction of temperature suitability and rainfall shows a stronger concentration in the highly endemic zone in West and Central Africa than the EVI.

**Fig 3:**
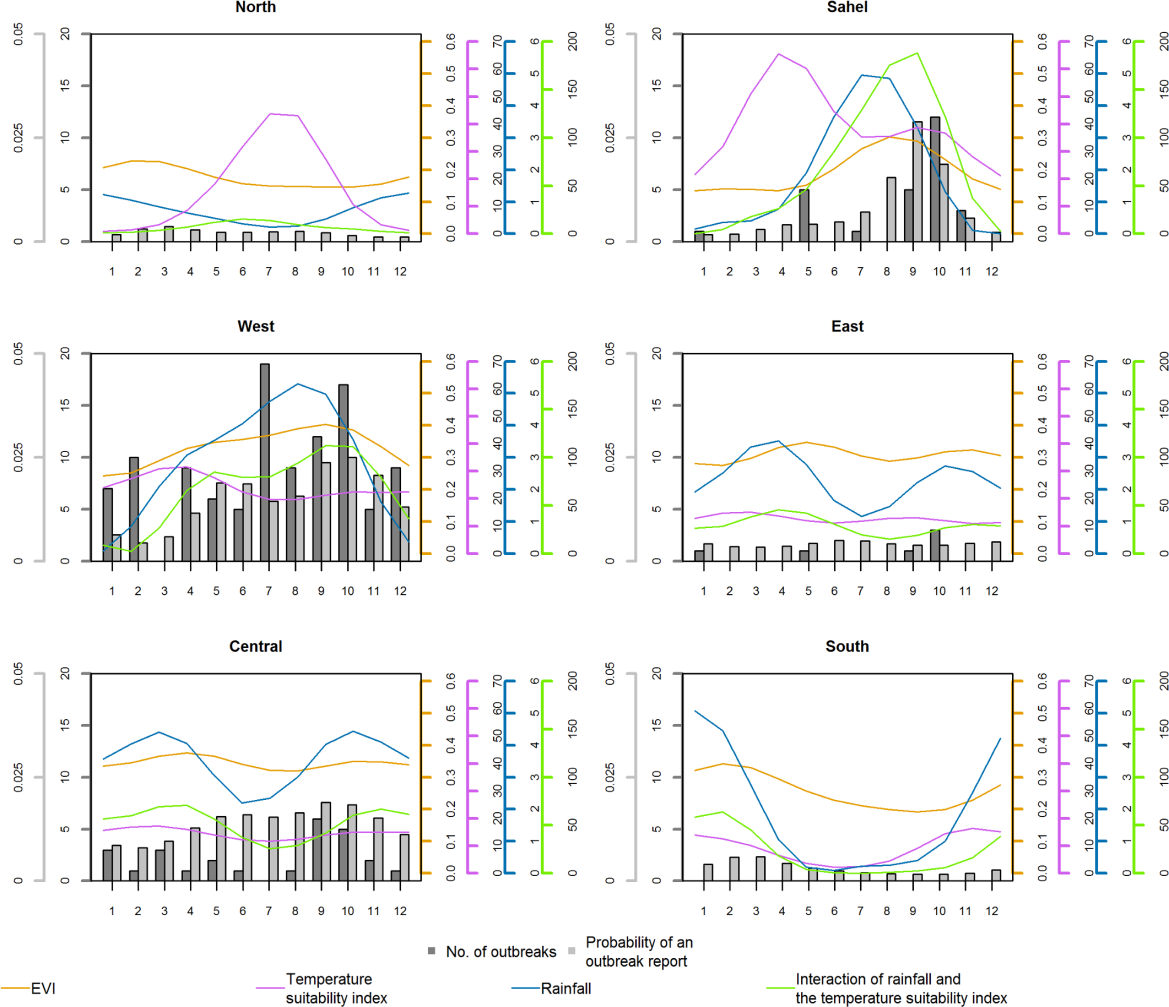
Seasonal patterns in YF reports and covariates by geographical region. Left axis (left to right): the mean monthly probability of YF reports and the number of YF reports. Right axis (left to right): monthly means of EVI, temperature suitability index, rainfall and the interaction of temperature suitability and rainfall. Colours of axes indicate covariate. See Fig 1 in S4 Text for classifications of different regions of Africa.

Fitting multilevel logistic regression models to time independent covariates (surveillance quality and population sizes) and monthly (environmental) covariate data produced seasonally variable predictions for the probability of YF reports across Africa with an AUC value of 0.81 (95% CI 0.79–0.84). The seasonality of YF reports, seasonal model predictions, the temperature suitability index, rainfall and EVI, differ between regions across Africa (Fig 3 and S1 Movie). The number of YF reports differs considerably between regions, but predominantly occur in September and October, with a secondary peak in spring/early summer across all regions, suggesting that there are two periods of heightened transmission annually. Seasonal variation in the EVI is low, with the largest differences being observed in the Sahel. This covariate has a significant effect on model fit (Fig in S2 Text). In YF endemic areas the temperature suitability index is fairly stable throughout the year while it drops to very low values in the respective winters in Northern and Southern Africa. Rainfall and EVI patterns are broadly similar to each other, with EVI typically lagging behind rainfall by approximately a month. These specific patterns differ across the continent with a single peak in the Sahel and West Africa, but with a bimodal pattern in Central and East Africa. The interaction of temperature suitability and rainfall mirrors the EVI throughout much of Africa, albeit at a lower magnitude in all but the Sahel.

**Fig 4:**
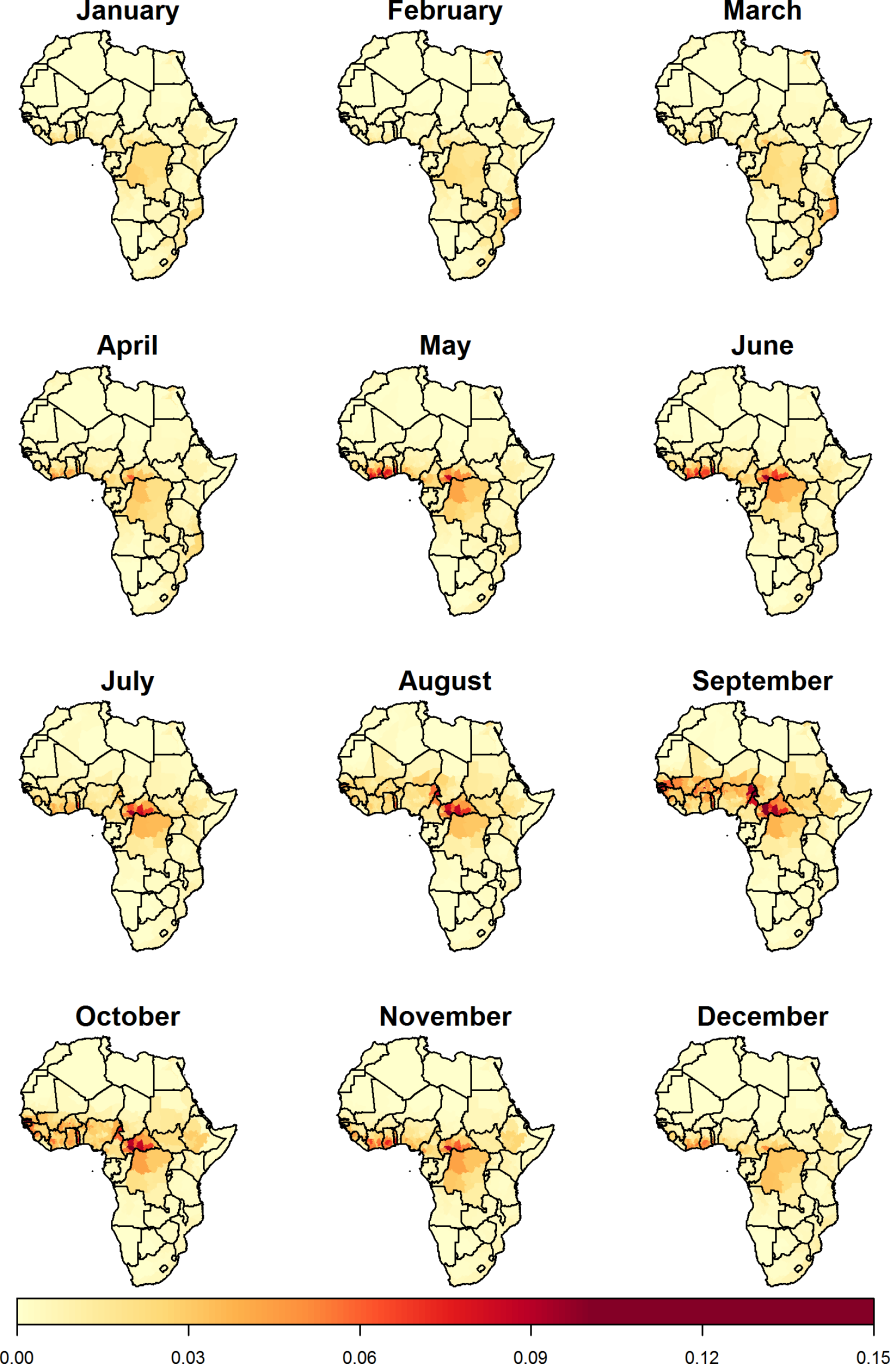
Seasonal model monthly predictions. Model predictions of the monthly YF report probabilities across Africa from the seasonal model. The AUC was 0.81 (0.78; 0.84).

The probability of a report varies throughout the year, with an oscillating pattern rolling northwards across the continent from March where it peaks in the Sahel during September, then returns southwards, resulting in biannual peaks of risk in West Africa with the risk extending further east during the main peak in October (Fig 3, Fig 4 and S1 Movie). The probability of YF reports, as predicted by the seasonal model varies geographically, with very low predictions in Northern, Southern and East Africa. The probability of a YF report in the Sahel is minimal throughout most of the year but rises sharply from around June to a peak in September, while in West Africa there is a substantial YF report probability throughout the year, apart from January to March where it is slightly lower. The seasonal variation of the probability of a YF report in Central Africa is synchronous to that in West Africa, although the amplitude of the variation is much reduced. As for the annual model (Fig 2), the probability of a report is relatively high in the Democratic Republic of the Congo due to the large population size in these provinces. For a given individual risk of infection, the probability of an report occurring is larger in a large underlying population, so this heightened probability reflects the aggregation level of populations more than the transmission intensity.

Aggregating the seasonal model predictions to a compound seasonal model gives very similar results to the annual model both in AUC and predictions, despite the magnitude of predictions being lower in the compound seasonal model (see S4 Text for further details).

In both the annual and the seasonal models the EVI was a significant coefficient in all models included in the weighted model, and is a significant predictor of model fit. For the weighted annual model, all contributing models also included the temperature suitability index, and for the weighted seasonal model, the interaction of the temperature suitability index and rainfall was included in all contributing models (S2 Text).

Leave-one-out cross validation of the annual, compound seasonal and seasonal model assessed the predictive performance of our models and found the AUC values of out-of-sample predictions were not significantly different from the AUC values based on full model predictions, suggesting the models were not affected by over-fitting (S5 Text). In addition to this, each of the 10 out-of-sample realisations had overlapping confidence intervals indicating the individual predictions were not significantly different.

## Discussion

In this study we developed a temperature suitability index for YFV transmission based on a mechanistic model of mosquito life history and YFV replication within the mosquito. We fitted multilevel logistic regression models to datasets of YF reports using the temperature suitability index as well as other relevant climatic variables to estimate monthly and annual probabilities of YF reports across mainland Africa at the province level. By using rainfall, the temperature suitability index, their interaction evaluated on a monthly timescale to capture synchronicity, the EVI (enhanced vegetation index), and factors related to case detection and human populations, we have explained a large amount of the geographic and temporal variation in the distribution of YF reports. While further variables associated with arbovirus transmission could potentially increase the specificity and sensitivity of the model, by focusing on a few well documented variables we serve to enhance the mechanistic understanding while still managing to explain a large amount of geographic and temporal heterogeneity. The model fit, as quantified by the AUC, is not significantly different between the compound seasonal and annual models despite the monthly model capturing seasonal trends in transmission in addition to the geographic heterogeneity without overfitting (S5 Text). The results show that areas with high annual probabilities of a report do not always possess the same risk throughout the year.

The 167 YF reports utilised were selected based on the availability of information on the location at the province level and the month during which the outbreak started. These reports rely on the accurate identification of YF cases, which can be problematic due to the presence of asymptomatic infections and diseases with similar symptoms [4, 45, 46]. In order to account for the inconsistencies between countries’ identification of cases, country-specific surveillance qualities were calculated using the reporting rate of suspected cases of YF for countries contributing to the YFSD and a constant incidence of syndrome “fever and jaundice”. For the remaining countries we assigned the mean surveillance quality value in the database to account for the level of under-reporting [3]. While the incidence of syndrome “fever and jaundice” is unlikely to be constant across the same region, the majority of cases highlighted by this will be caused by hepatitis and as such will not have the same variability geographically as a vector-borne disease such as YF.

Additionally, due to the timespan investigated (1971–2015) errors may be introduced in disease reporting by shifting political boundaries. While important to note, this is unlikely to result in substantial issues with geolocation of cases, particularly as for any cases where more detailed geographical information was available we mapped these using a consistent set of modern political boundaries.

The highest values of the temperature suitability index were found in areas with the highest temperatures, such as the Sahara, Sahel and parts of Southern Somalia (Fig 2E). While the temperature suitability index in these regions is highly suitable for the transmission of YF, the regions’ low levels of rainfall (Fig 2D) coupled with mosquitoes’ dependence on water for breeding lead to an inhospitable environment for mosquito reproduction. The necessity for both optimal temperatures and rainfall is depicted through the oscillating peaks in the probability of YF reports which closely follow the interaction of temperature suitability and rainfall (S1B and S1F Movie). The absence of this interaction offers an explanation for the lowered report predictions in Angola and Uganda, despite a historical presence of cases. Additionally, with respect to Angola and Uganda, it is important to note that by using covariates averaged over a period of years (2003–2006) we are capturing the typical pattern of seasonality, but neglecting inter-annual variability. This results in the de-emphasising of abnormal weather patterns and climate cycles such as El Niño Southern Oscillation, which have been found to affect vector-borne disease transmission [20, 47].

While the effect of temperature induced mortality on the temperature suitability index may be exaggerated in our parameterisation, temperatures in our dataset have a maximum of 38°C and so this does not affect the calculations when applied to our data. Additionally, we do not capture well the effect of low temperatures (<10°C) on mortality, but at these temperatures the extreme value of the EIP will dominate, resulting in a low temperature suitability index.

The interaction of the temperature suitability index and rainfall was significant in all models contributing to the combined seasonal model, lending credence to the idea that this interaction of rainfall and temperature is more important than either factor alone. However, the EVI was found in all the best-fitting annual and seasonal models (S2 Text). As continent wide covariates the temperature suitability and rainfall interaction is highly correlated with the EVI, and ranges from medium to high correlations at the regional level (S1 Text). Coupled with its high predictability, this suggests the EVI may account for the interaction of rainfall and the temperature suitability index, while providing additional information not captured by either, potentially due to the EVI quantifying a more complex relationship between temperature and environmental suitability than what is captured by their direct interaction. One explanation for this may be the EVI offering a more suitable proxy for the presence of standing water. While precipitation heavily influences the availability of standing water, ground permeability and the topographic redistribution of water additionally affect the presence of standing water, and so mosquito breeding sites. To more accurately predict the occurrence of YF, the availability of standing water and its interaction with temperature, rather than levels of precipitation, should be considered. To further increase accuracy, this could take into account anthropomorphic water storage and its influence on sustaining mosquito populations in the absence of naturally maintained water sources [48]. However, despite the worse performance of the interaction of temperature suitability and rainfall compared to the EVI as a predictor of YF reports, the former offers a mechanistic insight into the spatio-temporal variability and therefore enhances our understanding of the factors influencing YFV transmission intensity.

The temperature suitability index (Eq 2) assumes the inclusion of all necessary temperature dependent variables. However there are mechanisms that may be temperature dependent that we have not included due to the lack of suitable data, such as the vector to host ratio [11, 49] and the tendency to take multiple blood meals per gonotrophic cycle [50]. Regardless, though our temperature suitability index may only quantify part of the temperature dependence of transmission, we believe that it offers an adequate parameterisation. While this model has only investigated the role of *Ae*. *aegypti* as a vector, the principal vector of urban YF, within Africa there are over 20 species of *Aedes* that can transmit YF as well as members of *Eretmapodites* [51, 52]. Though there are likely to be some differences in climate suitability and vector competence between species, the underlying mechanisms are likely similar and any differences observed will be differences in model parameterisation. Therefore despite the model being calibrated for *Ae*. *aegypti*, it provides a good generalised estimate for all mosquito vectors, with the possibility of easy refinement for other species given relevant data. Furthermore, the framework could also be adapted to describe the seasonality of further mosquito-borne diseases, with the fewest adaptations necessary for other viruses transmitted by *Ae*. *aegypti*.

Despite climatic factors integral role in vector-borne disease transmission, they do not operate in a vacuum. Socioeconomic conditions such as informal housing and lack of sanitation greatly influence the presence of *A*. *aegypti* [53], creating conditions ideal for disease transmission. The importance of taking these factors into account is highlighted by our models’ low predictions in Luanda, where the 2015–2016 outbreak started, and suggests that the inclusion of measures of socioeconomic status may improve predictions of the geospatial occurrence of YF.

In contrast to earlier work on the distribution of burden of YF in Africa by Garske et al., (2014) [3], this body of work presents an initial simple framework for explaining YF report occurrence through a limited number of covariates, as well as exploring the role seasonal variation in these has on reports of YF. We believe that the work presented here is a valuable contribution in its present form as the limited number of covariates allows us to focus on enhancing the understanding of underlying processes using a robust statistical framework. The present study is therefore valuable on its own, but will also serve as an important stepping stone for future developments building upon the insights gained here with the addition of additional demographic and environmental variables, as well as the utilisation of a Bayesian methodology in line with work conducted by Garske et al., (2014)[3] and Bhatt et al., (2013)[54].

While research into the seasonality of vector-borne diseases has previously been undertaken [16, 24, 55], it had not been applied to YF and these findings represent an important first step in quantifying and understanding the seasonality of YF. This study has significant and far reaching consequences; the presence of both temporal and spatial aspects to transmission opens up the possibility of seasonally timed vector-borne disease control and the potential for the development of “early-warning” systems, which evaluate current or future climatic variables in order to predict periods of heightened transmission potential for YFV.

## Supporting information

S1 Text Correlations between covariates. (DOCX)

S2 Text Fitted model parameters and model inclusion. (DOCX)

S3 Text Receiver operating characteristic plots of models included in weighted models. (DOCX)

S4 Text Comparison of the annual and seasonal model predictions. (DOCX)

S5 Text Out-of-sample predictive ability. (DOCX)

S1 Table Annual models and their coefficient estimates (95% CIs). (DOCX)

S2 Table Seasonal models and their coefficient estimates (95% CIs). (DOCX)

S3 Table Model covariates for the annual model. (CSV)

S4 Table Model covariates for the seasonal model. (CSV)

S1 Movie Seasonal variation in covariates and predictions. A) Presence/absence of yellow fever reports (1971–2015) that fit inclusion criteria on a monthly scale, B) monthly probabilities of a yellow fever report, from 1971–2015, evaluated on a weekly time step, C) weekly mean EVI, D) weekly mean rainfall, E) weekly mean annual temperature suitability index, F) weekly mean interaction of temperature suitability index and rainfall delayed by one month.
(MP4)
