# Supplementary material for: The seasonal influence of climate and environment on yellow fever transmission across Africa
Source: PLoS Negl Trop Dis. 2018 Mar 15;12(3):e0006284. doi: 10.1371/journal.pntd.0006284 (PMC5854243; doi:10.1371/journal.pntd.0006284)
Supplement: S1 Text — (DOCX) [file pntd.0006284.s001.docx]

Correlations between covariates

Africa was divided into 6 regions based on ecological and climatic variables, with regions illustrated in SI Text 4.

The patterns of correlations between covariates differ between the different regions, with Northern Africa showing considerably different patterns while correlations between covariates are more similar between the sub-Saharan regions (Fig 1). In sub-Saharan Africa, the correlations between temperature suitability index and each of rainfall, the interaction between rainfall and the temperature suitability index, and EVI are fairly low in magnitude, while there are substantial positive correlations between the latter three covariates.

Rainfall and temperature suitability index are negatively correlated at a low value, apart from Southern Africa where there is a low positive correlation. The interaction of the temperature suitability index and rainfall and the temperature suitability index (Fig 1B) are only weakly correlated, with variable patterns across the continent. The EVI is negatively associated with the temperature suitability index (Fig 1C) with higher values of the temperature suitability index potentially indicating arid desert areas, unsuitable for plant growth. Low values of the temperature suitability index can also contain areas of low EVI which may relate to cold, mountainous terrain or to areas above 41 °C (Fig 1 main paper).

Rainfall and the interaction of the temperature suitability index and rainfall (Fig 1D) are highly correlated in all but Northern Eastern Africa. This may be due a lack of rainfall, but what rainfall does occur falls in the winter months where the temperature suitability index is very low.

EVI and rainfall (Fig 1E) are generally highly correlated across the continent as water availability limits plant growth. In Northern Africa high values of EVI with low values of rainfall may be explained by the Nile, which allows abundant plant growth despite very low levels of rainfall in the regions it passes through (Fig 2C in the main paper).

The correlation between the interaction of rainfall and the temperature suitability index and EVI (Fig 1F) is positive throughout Africa, albeit with some variation in magnitude. Low values of the interaction throughout Northern Africa are not mirrored in the EVI; and a similar pattern is found in Eastern Africa. Trends in the Sahel, Western, Central and Southern Africa are positive, though lower in Western Africa than the others. These trends are explained by the differences in the relative contributions of the temperature suitability index and rainfall to the interaction (Fig 1B and D).

While in some regions variables are highly correlated, these trends are not found in throughout, with correlations varying in magnitude and direction between different regions. This suggests that the variables capture different information, justifying the inclusion of all as potential covariates of yellow fever occurrence. This investigation also offers a possible explanation for the high predictability of EVI (Fig 3 and SI Table 1 and 2). The EVI has a moderate negative correlation with the temperature suitability index (apart from Southern Africa), and a moderate positive correlation with rainfall in all regions, suggesting that the EVI accounts for both rainfall and the temperature suitability index as a covariate, while providing additional information not captured by either. Furthermore, the differences in covariate correlations between different regions suggest that our assignment of countries to regions captures differences in environmental conditions.


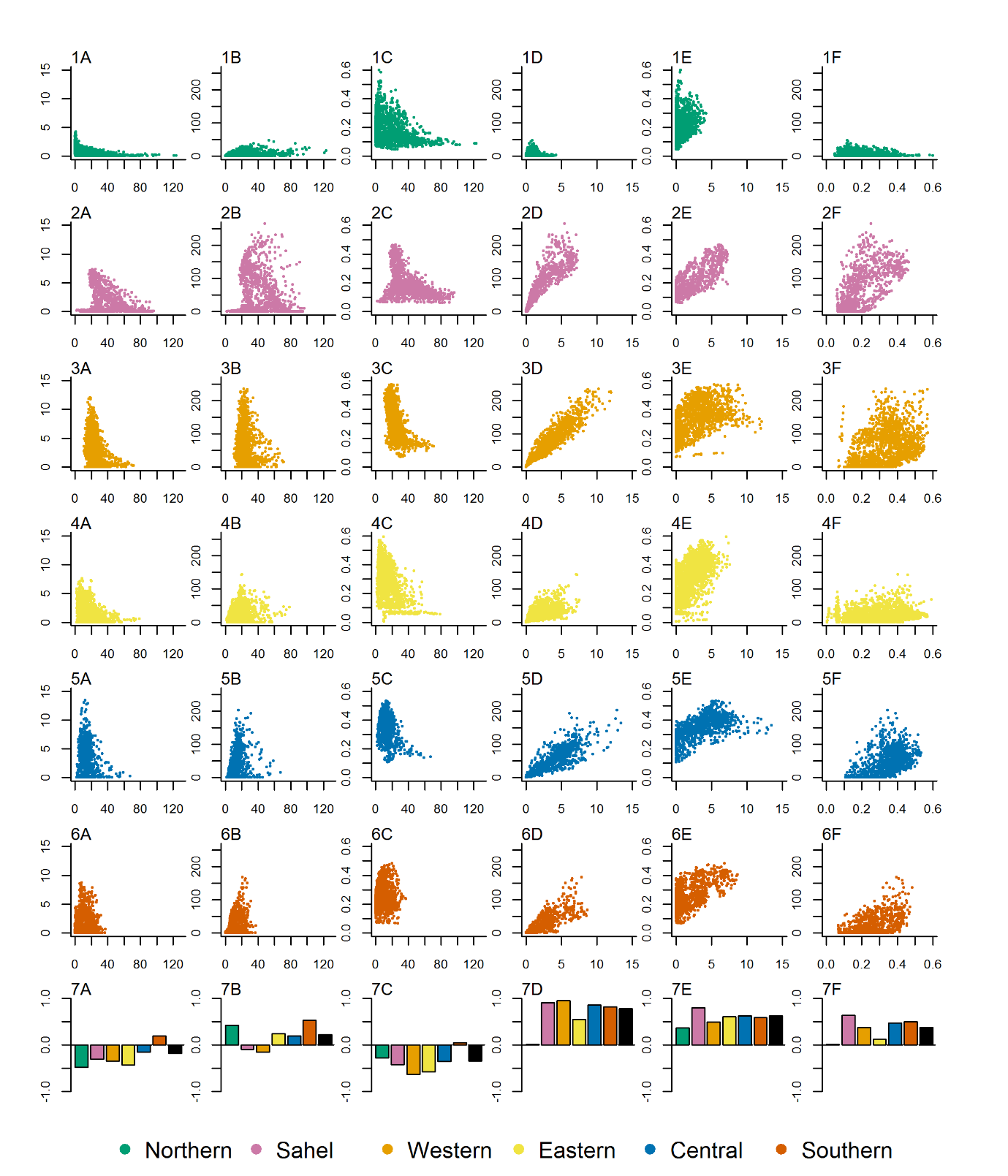


Fig 1. Covariates correlations by regions. Bivariate scatter plots of covariates by region as defined in Supplementary Fig 4, plotting one point per province and calendar month (averaged across years) for rows 1-6 and showing the correlation value of covariates in row 7. Column values are A) rainfall against the temperature suitability index, B) the interaction of rainfall and the temperature suitability index against the temperature suitability index, C) EVI against the temperature suitability index, D) the interaction of rainfall and the temperature suitability index against rainfall, E) EVI against rainfall, F) the interaction of rainfall and the temperature suitability index against the EVI.
