# Supplementary material for: The seasonal influence of climate and environment on yellow fever transmission across Africa
Source: PLoS Negl Trop Dis. 2018 Mar 15;12(3):e0006284. doi: 10.1371/journal.pntd.0006284 (PMC5854243; doi:10.1371/journal.pntd.0006284)
Supplement: S2 Text — (DOCX) [file pntd.0006284.s002.docx]

Fitted model parameters and model inclusion

We show the fitted model coefficients and the parameters from the combined model using Akaike weights based on the best fitting models as characterised by those models with an AIC value of less than 5 larger than the best fitting model (SI Fig 3 and SI Tables 1 and 2). Surveillance quality and log population were included in all models by design, while the four environmental covariates were not present in all models and were plotted as 0, without confidence intervals, when not included. For the combined model if a parameter was not included in a particular contributing model the parameter was given a value of 0 as this choice will counteract the bias away from 0 that can be found in quantitative model selection methods ([3](#_ENREF_3)). While the averaged coefficients were not used to derive the final predictions, the model predictions were weighted and averaged as stated in the main text, the weighted average of included model coefficients provide an insight into coefficient direction and magnitude.

For both weighted models the surveillance quality, log10 of population and the environmental covariates of EVI and temperature suitability index were significant. While in the seasonal model the interaction of the temperature suitability index with rainfall was additionally significant.


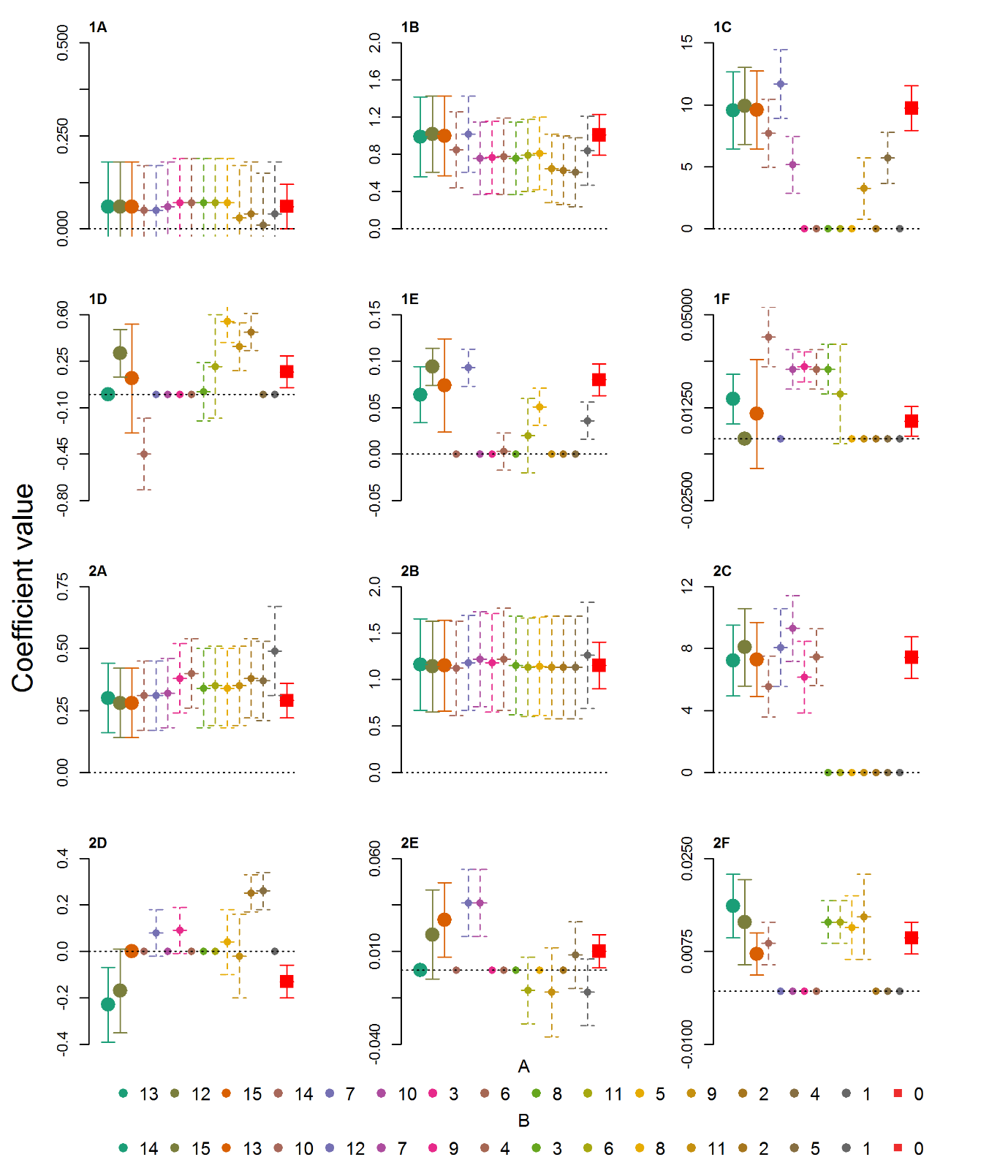


Fig 1. Coefficient values of models. The coefficient values of the A) surveillance quality, B) log of population, C) EVI (mean for the annual models and delayed by 1 month for the seasonal), D) rainfall, E) temperature suitability index F) interaction of the temperature suitability index and rainfall delayed by one month of 15 1) annual and 2) seasonal models ordered by AIC from left to right. The combined model coefficient value indicates the sum of included model akaike weighted coefficients and is depicted with a red square. Bars indicate 95% confidence intervals. Solid lines and enlarged points denote inclusion in the combined model, dashed lines and reduced points indicate exclusion. The legend is ordered by AIC from left to right for the 1) annual and 2) seasonal models with numbers referring to the model indices shown in SI Tables 1 and 2.
