# Supplementary material for: The seasonal influence of climate and environment on yellow fever transmission across Africa
Source: PLoS Negl Trop Dis. 2018 Mar 15;12(3):e0006284. doi: 10.1371/journal.pntd.0006284 (PMC5854243; doi:10.1371/journal.pntd.0006284)
Supplement: S3 Text — (DOCX) [file pntd.0006284.s003.docx]

Receiver Operating Characteristic plots of models included in weighted models

ROC curves of the models included in the combined models are shown in SI Fig 4 for both the annual and seasonal models. The area under the curve (AUC) values of included models show a high degree of similarity.


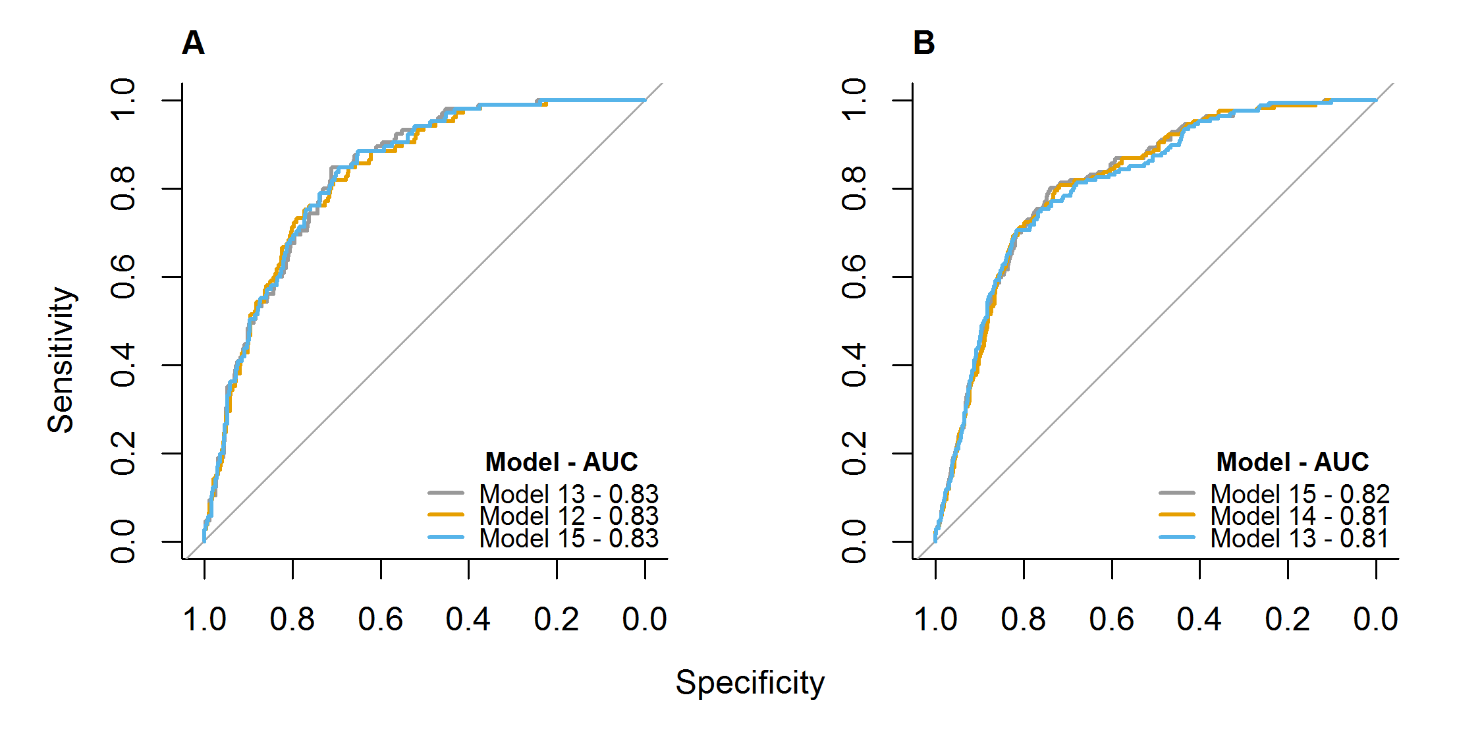


Fig 1. ROC curves. (A) Three annual models included in the annual combined model and (B) three seasonal models included in the seasonal combined model.
