# Supplementary material for: The seasonal influence of climate and environment on yellow fever transmission across Africa
Source: PLoS Negl Trop Dis. 2018 Mar 15;12(3):e0006284. doi: 10.1371/journal.pntd.0006284 (PMC5854243; doi:10.1371/journal.pntd.0006284)
Supplement: S4 Text — (DOCX) [file pntd.0006284.s004.docx]

Comparison of the annual and seasonal model predictions

Generally, the annual and compound seasonal models show similar geographical trends, with AUC values not significantly different (see SI Text, section 5), with a high correlation between annual and seasonal model predictions (0.94), see SI Fig 5. However, the model predictions of the annual model are consistently higher than those of the compound seasonal model for reasons not yet characterised. We calculated the expected number of provinces with yellow fever reports as a sum of the model predictions across provinces for both the annual and compound seasonal models, assuming an underlying inhomogeneous Bernoulli process, and compared this to the number of provinces with outbreaks observed in the data (SI Fig 6). Both models show high rates of agreement in the regions of low risk including North, East and South Africa but differ in the Sahel, West and Central Africa, where the annual model shows more accurate predictions in the Sahel and West Africa. Across Africa as a whole the annual model shows a better accuracy in predicting the number of reports in a year.


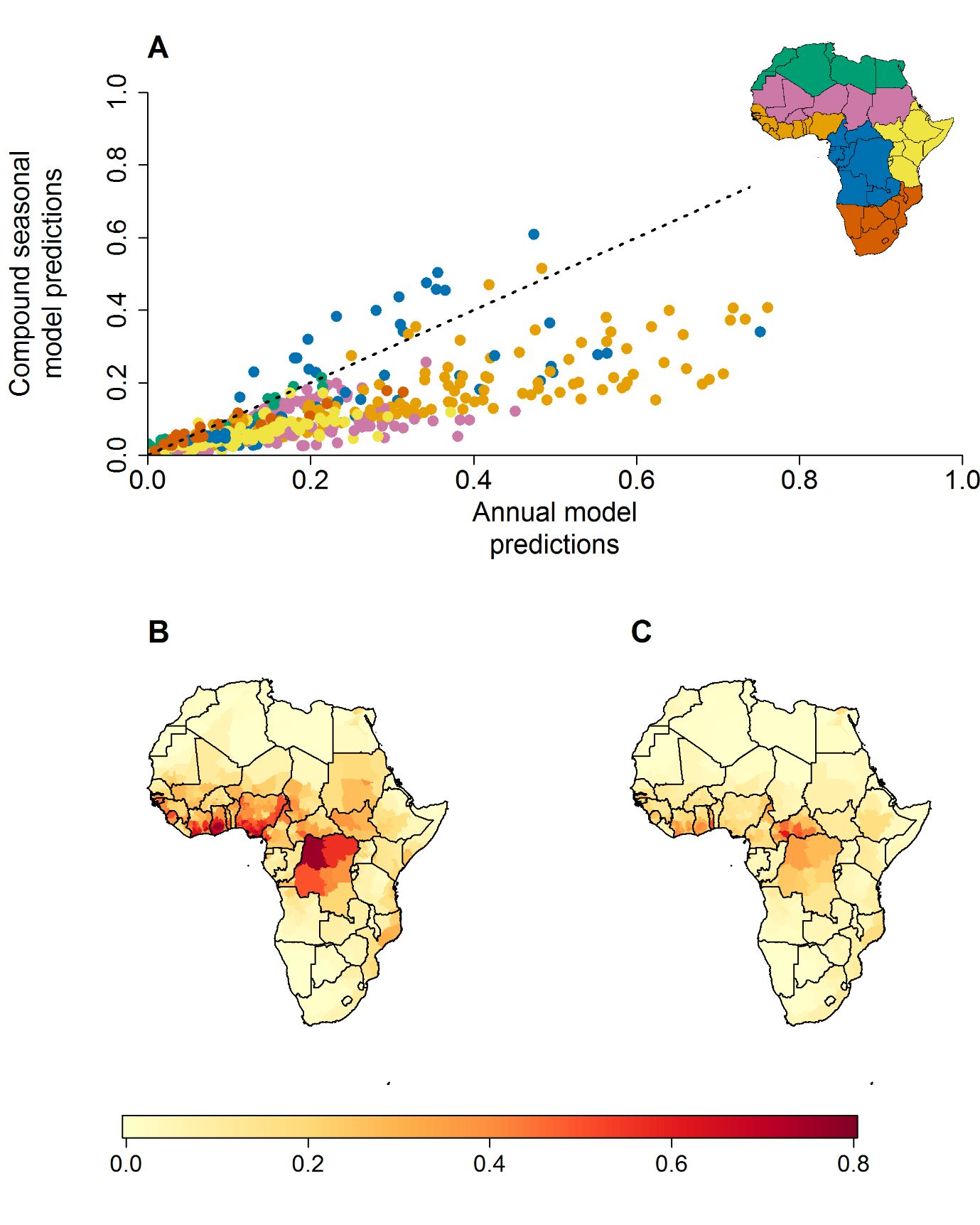


Fig 1. Annual and compound seasonal comparisons. A) Probabilities of yellow fever reports of compound seasonal compared with the annual model. The black dashed line indicates agreement between models. Colour of the dots indicates the region as shown in the inset map. B) Annual model probability of yellow fever reports (AUC = 0.83, 95% CI 0.80 – 0.87). C) Compound seasonal model probability of reports (AUC = 0.85, 95% CI 0.81 – 0.88).


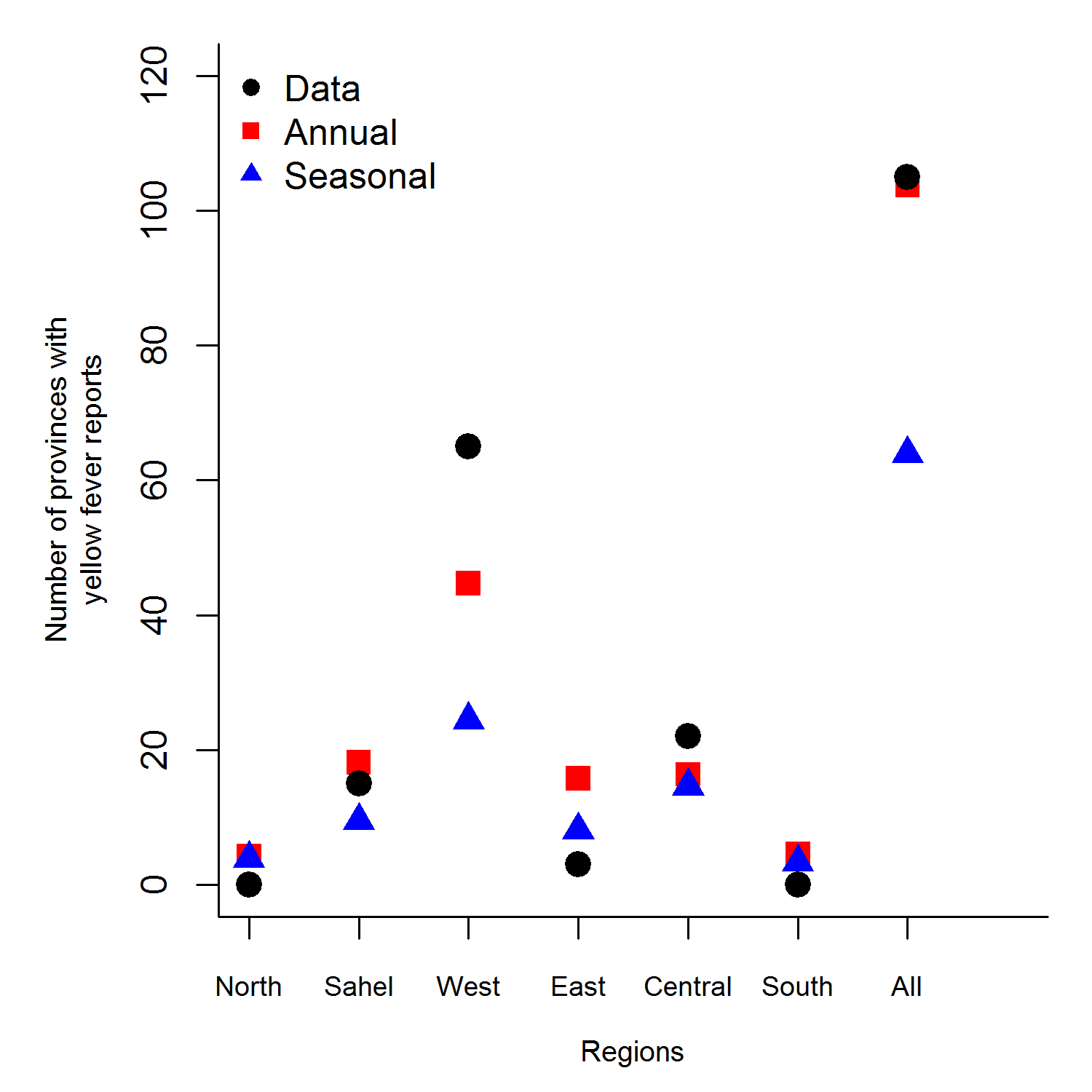


Fig 2. The expected and observed numbers of provinces with outbreak reports for different regions and across Africa. The black dots indicate the observed number of provinces with yellow fever reports, the red squares the number of provinces with reports expected under the annual model and the blue triangles the number of provinces with reports expected under the compound seasonal model.
