# Supplementary material for: The seasonal influence of climate and environment on yellow fever transmission across Africa
Source: PLoS Negl Trop Dis. 2018 Mar 15;12(3):e0006284. doi: 10.1371/journal.pntd.0006284 (PMC5854243; doi:10.1371/journal.pntd.0006284)
Supplement: S5 Text — (DOCX) [file pntd.0006284.s005.docx]

Out-of-sample predictive ability

In order to quantify the out-of-sample predictive ability of our models we conducted Leave-one-out cross validation (LOOCV) for the annual, compound seasonal and full seasonal models. This was done by splitting the dataset, by countries, into five segments and training the model on four of them. Once trained the model was extrapolated to the missing segment. This was repeated for each of the segments which were combined to produce predictions for all of mainland Africa. This process was repeated 10 times to generate 10 different compositions of the 5 subsets. For each province, the average predictions across the 10 realisations were used to calculate the AUC of their ROC curves in order to contrast the out-of-sample predictions against the full models.

The annual model, compound seasonal and full seasonal model all show high rates of correlation between the out-of-sample predictions and the predictions of the models fitted to the full dataset (0.83, 0.94 and 0.87 respectively). The confidence intervals of the AUC for the full model fit and the out-of-sample predictions overlap for all three models with 0.83 (0.80; 0.87) and 0.79 (0.75; 0.83) for the annual model, 0.85 (0.81; 0.88) and 0.80 (0.76; 0.84) for the compound seasonal model, and 0.81 (0.79; 0.84) and 0.77 (0.74; 0.81) for the full seasonal model. This indicates that the out-of-sample predictive ability of the models as assessed, is very high.


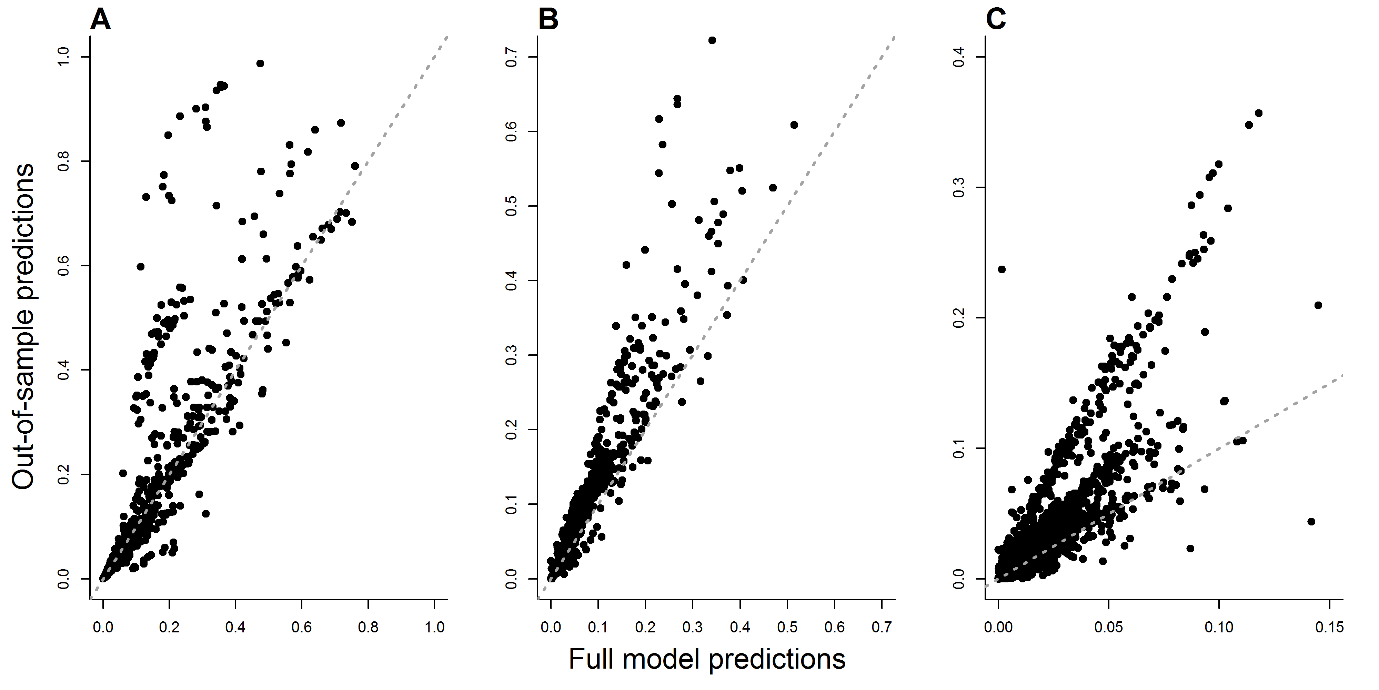


Fig 1. Cross validation of the three combined models used. Out of sample model predictions against the predictions of the model fitted to all data for A) the annual model, B) the compound seasonal model and C) the full seasonal model. The grey dashed line indicates agreement between out-of-sample and full model predictions.
