# Supplementary material for: The seasonal influence of climate and environment on yellow fever transmission across Africa
Source: PLoS Negl Trop Dis. 2018 Mar 15;12(3):e0006284. doi: 10.1371/journal.pntd.0006284 (PMC5854243; doi:10.1371/journal.pntd.0006284)
Supplement: S1 Table — (DOCX) [file pntd.0006284.s006.docx]

| **S1 Table. Annual models and their coefficient estimates (95% CIs)** | | | | | | | | | | | |
| --- | --- | --- | --- | --- | --- | --- | --- | --- | --- | --- | --- |
| Model | **Intercept** | **Surveillance quality** | **Log of population** | **EVI** | **Rainfall** | **Temperature suitability index** | **Interaction of temperature suitability and rainfall** | **AUC** | **AIC** | **Contribution to weighted model (%)** | **No. of parameters** |
| 13* | -12.51  (-15.72; -9.30) | 0.06  (-0.06; 0.18) | 0.99  (0.56; 1.42) | 9.55 (6.45; 12.65) | - | 0.064  (0.034; 0.094) | 0.016  (0.006; 0.026) | 0.83 | 478.7 | 47 | 6 |
| 12* | -13.46  (-16.62; -10.30) | 0.06  (-0.06; 0.18) | 1.02  (0.61; 1.43) | 9.92  (6.82; 13.02) | 0.31  (0.13; 0.49) | 0.094  (0.074; 0.114) | - | 0.83 | 479.4 | 33 | 6 |
| 15* | -12.85  (-16.24; -9.46) | 0.06  (-0.06; 0.18) | 1.00  (0.57; 1.43) | 9.59  (6.45; 12.73) | 0.12  (-0.29; 0.53) | 0.074  (0.024; 0.124) | 0.01  (-0.012; 0.032) | 0.83 | 480.5 | 20 | 7 |
| 14 | -9.79  (-12.48; -7.10) | 0.05  (-0.07; 0.17) | 0.85  (0.44; 1.26) | 7.71  (4.97; 10.45) | -0.45  (-0.72; -0.18) | - | 0.041  (0.029; 0.053) | 0.83 | 487.6 | 0 | 6 |
| 7 | -13.18  (-16.3; -10.06) | 0.05  (-0.07; 0.17) | 1.02  (0.61; 1.43) | 11.68  (8.9; 14.46) | - | 0.093  (0.073; 0.113) | - | 0.82 | 488.5 | 0 | 5 |
| 10 | -8.99  (-11.56; -6.42) | 0.06  (-0.06; 0.18) | 0.76  (0.37; 1.15) | 5.17  (2.88; 7.46) | - | - | 0.028  (0.020; 0.036) | 0.82 | 496.1 | 0 | 5 |
| 3 | -7.57  (-10.00; -5.14) | 0.07  (-0.05; 0.19) | 0.77  (0.38; 1.16) | - | - | - | 0.029  (0.023; 0.035) | 0.79 | 514.3 | 0 | 4 |
| 6 | -7.7  (-10.25; -5.15) | 0.07  (-0.05; 0.19) | 0.78  (0.37; 1.19) | - | - | 0.003  (-0.017; 0.023) | 0.028  (0.020; 0.036) | 0.79 | 516.2 | 0 | 5 |
| 8 | -7.56  (-9.99; -5.13) | 0.07  (-0.05; 0.19) | 0.76  (0.37; 1.15) | - | 0.02  (-0.20; 0.24) | - | 0.028  (0.018; 0.038) | 0.79 | 516.2 | 0 | 5 |
| 11 | -8.2  (-10.90; -5.50) | 0.07  (-0.05; 0.19) | 0.79  (0.40; 1.18) | - | 0.21  (-0.18; 0.60) | 0.02  (-0.020; 0.060) | 0.018  (-0.002; 0.038) | 0.79 | 517.2 | 0 | 6 |
| 5 | -8.98  (-11.49; -6.47) | 0.07  (-0.05; 0.19) | 0.81  (0.42; 1.20) | - | 0.55  (0.39; 0.71) | 0.051  (0.031; 0.071) | - | 0.78 | 518.8 | 0 | 5 |
| 9 | -7.41  (-9.78; -5.04) | 0.03  (-0.11; 0.17) | 0.65  (0.28; 1.02) | 3.25  (0.76; 5.74) | 0.36  (0.18; 0.54) | - | - | 0.71 | 548.0 | 0 | 5 |
| 2 | -6.65  (-8.88; -4.42) | 0.04  (-0.10; 0.18) | 0.63  (0.26; 1.00) | - | 0.47  (0.33; 0.61) | - | - | 0.70 | 552.4 | 0 | 4 |
| 4 | -7.02  (-9.39; -4.65) | 0.01  (-0.13; 0.15) | 0.61  (0.24; 0.98) | 5.72  (3.66; 7.78) | - | - | - | 0.69 | 561.7 | 0 | 4 |
| 1 | -7.41  (-9.8; -5.02) | 0.04  (-0.10; 0.18) | 0.84  (0.47; 1.21) | - | - | 0.036  (0.016; 0.056) | - | 0.70 | 571.0 | 0 | 4 |
| Weighted model | -13.02  (-14.69; -11.35) | 0.06  ( 0.00; 0.12) | 1.01  (0.79; 1.23) | 9.73  (7.93; 11.53) | 0.17  (0.05; 0.29) | 0.080  (0.063; 0.097) | 0.007  (0.001; 0.013) | 0.83 | NA | 0 | 7 |
| Models indicated with an asterisk (*) and emboldened were used in the final combined model. A dash (-) indicates that covariate was not included in the model. Models are ordered by AIC value with the model with the smallest value at the top. | | | | | | | | | | | |
