# Supplementary material for: The seasonal influence of climate and environment on yellow fever transmission across Africa
Source: PLoS Negl Trop Dis. 2018 Mar 15;12(3):e0006284. doi: 10.1371/journal.pntd.0006284 (PMC5854243; doi:10.1371/journal.pntd.0006284)
Supplement: S2 Table — (DOCX) [file pntd.0006284.s007.docx]

| **S2 Table. Seasonal models and their coefficient estimates (95% CIs)** | | | | | | | | | | | |
| --- | --- | --- | --- | --- | --- | --- | --- | --- | --- | --- | --- |
| Model | **Intercept** | **Surveillance quality** | **Log of population** | **EVI** | **Rainfall** | **Temperature suitability index** | **Interaction of temperature suitability and rainfall** | **AUC** | **AIC** | **Contribution to combined model (%)** | **No. of parameters** |
| 15* | -14.94 (-18.19; -11.69) | 0.28 (0.14; 0.42) | 1.14 (0.65; 1.63) | 8.08 (5.59; 10.57) | -0.17 (-0.35; 0.01) | 0.019 (-0.005; 0.043) | 0.013  (0.005; 0.021) | 0.82 | 1374.2 | 40 | 7 |
| 14* | -14.44 (-17.62; -11.26) | 0.30 (0.16; 0.44) | 1.16 (0.67; 1.65) | 7.22 (4.93; 9.51) | -0.23 (-0.39; -0.07) | - | 0.016  (0.010; 0.022) | 0.81 | 1374.2 | 40 | 6 |
| 13* | -15.11 (-18.38; -11.84) | 0.28 (0.14; 0.42) | 1.15 (0.66; 1.64) | 7.29 (4.90; 9.68) | - | 0.027 (0.007; 0.047) | 0.007  (0.003; 0.011) | 0.81 | 1375.6 | 20 | 6 |
| 10 | -14.04 (-17.23; -10.85) | 0.31 (0.17; 0.45) | 1.12 (0.61; 1.63) | 5.55 (3.59; 7.51) | - | - | 0.009  (0.005; 0.013) | 0.80 | 1379.8 | 0 | 5 |
| 12 | -15.68 (-19.01; -12.35) | 0.31 (0.17; 0.45) | 1.18 (0.67; 1.69) | 8.06 (5.55; 10.57) | 0.08 (-0.02; 0.18) | 0.036 (0.018; 0.054) | - | 0.81 | 1383.3 | 0 | 6 |
| 7 | -16.11 (-19.46; -12.76) | 0.32 (0.18; 0.46) | 1.22 (0.71; 1.73) | 9.30 (7.18; 11.42) | - | 0.036 (0.018; 0.054) | - | 0.81 | 1384.3 | 0 | 5 |
| 9 | -14.66 (-18.13; -11.19) | 0.38 (0.24; 0.52) | 1.18 (0.63; 1.73) | 6.16 (3.85; 8.47) | 0.09 (-0.01; 0.19) | - | - | 0.79 | 1394.0 | 0 | 5 |
| 4 | -15.13 (-18.64; -11.62) | 0.40 (0.26; 0.54) | 1.22 (0.67; 1.77) | 7.45 (5.61; 9.29) | - | - | - | 0.79 | 1395.1 | 0 | 4 |
| 3 | -13.02 (-16.35; -9.69) | 0.34 (0.18; 0.50) | 1.15 (0.62; 1.68) | - | - | - | 0.013  (0.009; 0.017) | 0.76 | 1408.6 | 0 | 4 |
| 6 | -12.76 (-16.11; -9.41) | 0.35 (0.19; 0.51) | 1.13 (0.6; 1.66) | - | - | -0.011 (-0.029; 0.007) | 0.013  (0.009; 0.017) | 0.76 | 1409.2 | 0 | 5 |
| 8 | -12.98 (-16.29; -9.67) | 0.34 (0.18; 0.50) | 1.14 (0.61; 1.67) | - | 0.04 (-0.10; 0.18) | - | 0.012  (0.006; 0.018) | 0.76 | 1410.2 | 0 | 5 |
| 11 | -12.74 (-16.09; -9.39) | 0.35 (0.19; 0.51) | 1.13 (0.58; 1.68) | - | -0.02 (-0.20; 0.16) | -0.012 (-0.036; 0.012) | 0.014  (0.006; 0.022) | 0.76 | 1411.1 | 0 | 6 |
| 2 | -13.12 (-16.53; -9.71) | 0.38 (0.22; 0.54) | 1.13 (0.58; 1.68) | - | 0.25 (0.17; 0.33) | - | - | 0.74 | 1420.1 | 0 | 4 |
| 5 | -13.20 (-16.57; -9.83) | 0.37 (0.21; 0.53) | 1.13 (0.58; 1.68) | - | 0.26 (0.18; 0.34) | 0.008 (-0.010; 0.026) | - | 0.75 | 1421.3 | 0 | 5 |
| 1 | -13.21 (-16.78; -9.64) | 0.49 (0.31; 0.67) | 1.26 (0.69; 1.83) | - | - | -0.012 (-0.030; 0.006) | - | 0.70 | 1462.7 | 0 | 4 |
| Weighted model | -14.81 (-16.46; -13.16) | 0.29 (0.22; 0.36) | 1.15 (0.90; 1.40) | 7.42 (6.07; 8.77) | -0.13 (-0.20; -0.06) | 0.010 (0.001; 0.019) | 0.010  (0.007; 0.013) | 0.81 | NA | 0 | 7 |
| Models indicated with an asterisk (*) and emboldened were used in the final combined model. A dash (-) indicates that covariate was not included in the model. Models are ordered by AIC value with the model with the smallest value at the top. | | | | | | | | | | | |
